# Experimental Evaluation of Dry Powder Inhalers during Inhalation and Exhalation Using a Model of the Human Respiratory System (xPULM™)

**DOI:** 10.3390/pharmaceutics14030500

**Published:** 2022-02-24

**Authors:** Richard Pasteka, Lara Alina Schöllbauer, Joao Pedro Santos da Costa, Radim Kolar, Mathias Forjan

**Affiliations:** 1 Department Life Science Engineering, University of Applied Sciences Technikum Wien, Höchstaedtplatz 6, 1200 Vienna, Austria; lara.schoellbauer@technikum-wien.at (L.A.S.); santosd@technikum-wien.at (J.P.S.d.C.); mathias.forjan@technikum-wien.at (M.F.); 2 Department of Biomedical Engineering, Brno University of Technology, Technicka 3058, 616 00 Brno, Czech Republic; kolarr@vutbr.cz

**Keywords:** dry powder inhaler resistance, inspiratory flow rate, inspiratory pressure, aerosol particle deposition, mechanical upper airway model, optical aerosol spectrometry, biomedical engineering

## Abstract

Dry powder inhalers are used by a large number of patients worldwide to treat respiratory diseases. The objective of this work is to experimentally investigate changes in aerosol particle diameter and particle number concentration of pharmaceutical aerosols generated by four dry powder inhalers under realistic inhalation and exhalation conditions. To simulate patients undergoing inhalation therapy, the active respiratory system model (xPULM™) was used. A mechanical upper airway model was developed, manufactured, and introduced as a part of the xPULM™ to represent the human upper respiratory tract with high fidelity. Integration of optical aerosol spectrometry technique into the setup allowed for evaluation of pharmaceutical aerosols. The results show that there is a significant difference (*p* < 0.05) in mean particle diameter between inhaled and exhaled particles with the majority of the particles depositing in the lung, while particles with the size of (>0.5 μm) are least influenced by deposition mechanisms. The fraction of exhaled particles ranges from 2.13% (HandiHaler^®^) over 2.94% (BreezHaler^®^), and 6.22% (Turbohaler^®^) to 10.24% (Ellipta^®^). These values are comparable to previously published studies. Furthermore, the mechanical upper airway model increases the resistance of the overall system and acts as a filter for larger particles (>3 μm). In conclusion, the xPULM™ active respiratory system model is a viable option for studying interactions of pharmaceutical aerosols and the respiratory tract regarding applicable deposition mechanisms. The model strives to support the reduction of animal experimentation in aerosol research and provides an alternative to experiments with human subjects.

## 1. Introduction

According to the report on the global impact of respiratory disease published by the Forum of International Respiratory Societies from 2017 [1] approximately 65 million people globally suffer from mild to severe chronic obstructive pulmonary disease (COPD) and 334 million people suffer from asthma. In conjunction with acute lower respiratory tract infections, these diseases are among the most prevalent severe illnesses and causes of death [1]. Based on Eurostat statistics [2] from 2016, diseases of the respiratory system accounted for approximately 7.5% of all deaths in the former EU-27. Targeted delivery of pharmaceuticals directly into the affected part of the respiratory region via inhalation drug therapy is crucial for managing cases of obstructive respiratory diseases [3].

Inhalation therapeutic devices can be categorized into four main types, including nebulizers, pressurized-metered dose inhalers (pMDI), soft mist inhalers (SMI), and dry powder inhalers (DPI) [3]. In terms of units sold in 2014, pMDIs and DPIs constitute the majority of devices for inhalation drug delivery [4]. For this reason, this article focuses purely on the evaluation of DPIs. In contrast to pMDIs, DPIs work with larger lactose particles carrying the active substance and are environmentally preferable due to the absence of hydrofluorcarbons [5]. Nevertheless, DPIs require a minimum peak flow during inhalation, created by the patient, to detach and propel the aerosol in direction of the lung regions. The lack of required synchronity between activation and inhalation of the DPI is reducing a potential source of misuse [6]. However, other errors such as loading and priming the DPI for use or exhalation into the DPI before the inhalation step are present and have a significant negative effect on the delivered dose [6,7]. The optimum flow profile varies for the currently available DPIs and may lead to a suboptimal delivered dose for the patients [6]. Most recently developed DPIs only deliver a low dose of medication while the users have to be able to create a minimum inspiratory flow and have the cognitive ability to properly operate the DPI [8]. This is accompanied by the need for an adequate lung volume of the user, therefore excluding children below the age of 5 years [8]. Considering only a single peak inspiratory flow rate (PIFR) value as the main criterion for determining the capability of the patient to use an inhaler efficiently may be an insufficient criterion as the DPIs vary in their design and resistance to airflow [9]. While several available studies evaluating DPIs focus mainly on inspiratory flow rate [10,11,12,13,14] a more suitable criteria has proved to be ensuring a sufficient pressure drop of ≥1 kPa across the device. Inhalation under these conditions leads to delivery of an adequate pharmaceutical dose to the lungs [9]. Focusing on the pressure drop across the device could help prevent exclusion of patients from DPI usage due to insufficient or excessive peak inspiratory flow rate. Both have been shown to negatively impact pulmonary drug delivery [10,15]. Therefore, the pressure drop over the DPI has been taken as the main evaluation criterion for successful inhalation processes for this work.

In vitro pharmaceutical aerosol test systems often include either sample collection tubes or cascade impactors, such as the Andersen nonviable impactor, or the Next Generation Impactor to collect the particles for classification [16,17,18]. The results using such systems provide insights about the properties of the inhaled aerosol, such as the sizes of the inhaled particles and the deposition fraction, which can be used for comparison with radionuclide imaging studies [19] or to validate in silico dosimetry models [20,21,22]. Cascade impactors consist of stages, each containing impaction plates which represent obstacles for an incoming airstream [23]. These plates create an abrupt bend in the airstream causing the particles, whose inertia exceeds a cutoff size, to deposit [24]. Due to the operating principle of cascade impactors and sample collection tubes, evaluation of aerosol during consecutive inhalation and exhalation is not feasible [25]. The aerosol particles deposit on the impaction plates during inhalation and are consequently not present in the exhalatory airstream. For this reason, optical aerosol spectrometry was utilized in this work allowing for evaluation of particles within both inhalation and exhalation airstream.

Pulmonary drug delivery is based on the primary mechanisms of aerosol deposition, which are defined as inertial impaction, gravitational sedimentation, Brownian diffusion, turbulent deposition, electrostatic precipitation, and interception [26]. The effect of deposition mechanisms on aerosol particles depends on the particle characteristics such as particle size, overall size distribution, shape, composition, surface characteristics, and charge [27]. Moreover, the processes resulting from molecular transfer between particles and their respective surrounding gas are nucleation, condensation, evaporation hygroscopicity, and coagulation [28]. Inhalation drug therapy aims at targeted delivery of pharmaceuticals into the lung. The inhaled particles must overcome filtration mechanisms in the upper airways causing them to deposit within this region [29]. The deposition mechanism occurring mainly in the upper airways is inertial impaction affecting mostly large particles (>2–5 μm) with a strong dependency on the airflow rate. The deposition in this region of the respiratory tract results from direction changes of flow when the particles deviate from the streamline and collide with the airway walls. The probability of such deviation can be described by the Stoke’s number where particle diameter, carrier gas viscosity, and airway diameter are used to calculate the probability of deposition [30]. In the respiratory tract, gravitational sedimentation of particles in the size range of (>1–8 μm), refers to the settling of particles under the influence of gravity. Brownian diffusion results from random motion and the collision of the particles with the carrier gas molecules. The effect of mutual repulsion due to electric charges concerning the inhaled particles is defined as electrostatic precipitation. The described mechanisms arise mostly in the upper and conducting airway region of the respiratory tract, whereas diffusion and electrostatic precipitation is also taking place in the acinus region of the pulmonary system for particles <3 μm. [26]

The objective of this work is to experimentally investigate changes in aerosol particle diameter and particle number concentration of pharmaceutical aerosols under realistic inhalation and exhalation conditions, resulting in a calculated lung deposition. The active respiratory system model (xPULM) used in this work includes two core elements: a computed-tomography (CT)-derived mechanical upper airway model (UAM) and a primed porcine lung serving as a human lung equivalent. This setup is used to represent a patient undergoing inhalation therapy. In contrast to widely spread measurement setups, this work integrates an optical aerosol spectrometer for inhalation and exhalation measurements to eliminate the drawbacks introduced by cascade impactors [25,31]. To cover a wide spectrum of devices used in clinical practice, four commonly used DPIs are investigated. Instead of focusing on PFIR, the focus was put on reaching a pressure drop of at least (P_DROP_ ≥ 1 kPa) for all inhalers. This article aims to provide an alternative respiratory model suitable to reduce animal experimentation in aerosol research. Furthermore, the work aspires to mitigate the shortage of experimental data, viable to substitute demanding and constrictive experiments with human subjects. Moreover, the experimental setup including the xPULM™ model, can be seen as a basis for an alternative to animal testing, as the porcine lung, included in this trial, was salvaged from an abattoir.

## 2. Materials and Methods

### 2.1. Measurement Setup and Procedure

**Figure 1:**
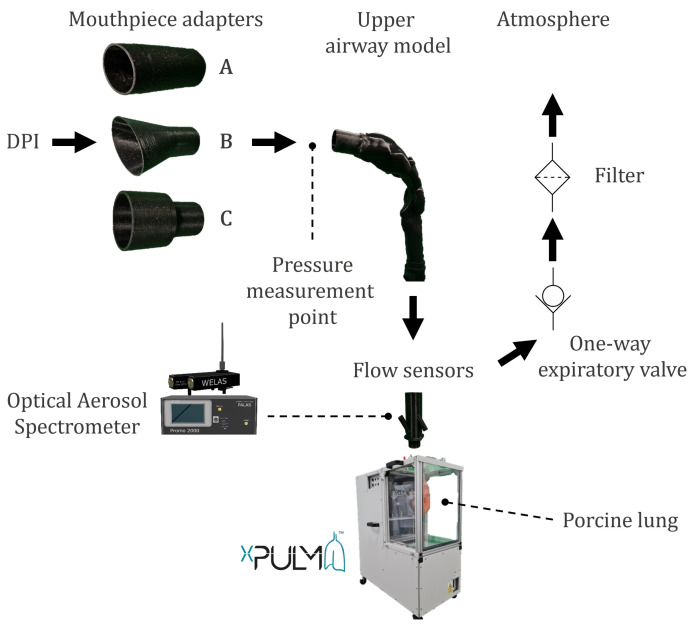
The measurement setup for respiration measurements consisting of mouthpiece adapters for (A) BreezHaler^®^, (B) Ellipta^®^, HandiHaler^®^, (C) Turbohaler^®^, the mechanical UAM derived from CT examinations, and the optical aerosol spectrometer used to characterize the aerosol particles and the active model of the human respiratory system xPULM™ with the porcine lung.

The following two measurement trials were conducted during this study: (A) characterization measurements and (B) respiration measurements. To assess the particles generated by the DPIs, characterization measurements were performed using a simple connection element to the respiratory model xPULM™. This connector is characterized by a simplified version of the human laryngeal space in the form of a 90-degree bend and includes a sampling nozzle. This aerosol sampling point is in line with the inhalatory airstream to ensure isoaxial aerosol sampling. Moreover, the control loop of the optical aerosol spectrometer maintains a constant sampling flow, regardless of the inhalation flow profile. The active model of the human respiratory system, xPULM™, was used with polymer breathing bags, to simulate the inhalation effort of a patient during particle characterization measurements. In the second step, respiration measurements (see Figure 1) were conducted to investigate changes in aerosol particle diameter and particle number concentration during inhalation and exhalation. For this purpose, a primed porcine lung was used as a anatomically realistic lung equivalent. The porcine lung has been proved to be a suitable model of the anatomy of the human lung [32] and has been used in previous studies to research the pathogenesis of diseases such as cystic fibrosis [33]. The lung equivalent was connected to a mechanical UAM which was rapidly manufactured using 3D-printing techniques. The UAM is based on a clinically annotated CT examination of a healthy subject. In contrast to the characterisation measurements, sampling took place at the lower end of the mechanical UAM to assess the influence of its geometry on the measured values. The DPIs were mounted to the UAM using custom mouthpiece adapters to ensure airtight connection.

The measurement procedure of the respiration measurements consists of three phases (i) inhalation, (ii) breath-hold, and (iii) exhalation. Inhalation with maximum effort was simulated until the pressure drop across the DPI, measured with the FlowAnalyser PF-300 (IMT Analytics, Switzerland), reached at least the recommended pressure drop of ≥1 kPa [9]. However, if achieveable, a pressure drop of 4 kPa was targeted [34]. The driving force of the inhalation was terminated when the peak value of the pressure drop was reached. However, inhalation continued briefly due to inertia and compliance of the lung equivalent. Inhaler-specific inhalation profiles were recorded using mass flow sensors SFM3300-AW (Sensirion, Switzerland).

All measurements were performed under laboratory conditions and environmental parameters were recorded. The results were adjusted for the recorded background aerosol load. After each measurement trial, breathing simulation was run until the background load was reached.

The inhalation manoeuvre was followed by a 5 s breath-hold period prior to slow and steady exhalation at a flow of 30 L/min for the duration of 6 s. For each tested DPI, the measurements were repeated 12 times (*n* = 12). The in-/exhalation airstream was sampled by the optical aerosol spectrometer Promo 2000 (PALAS, Karlsruhe, Germany) connected to a white light aerosol sensor Welas 2070 (PALAS, Germany) with a constant flow rate of 5 L/min. The sensor is capable of measuring particles in the range of 0.2 μm to 10 μm.

### 2.2. Model of the Human Respiratory System

The active model of the human respiratory system xPULM™ has been used in this study. The xPULM™ replicates human breathing efforts exerted during the use of DPIs. Fundamental respiratory characteristics (e.g., flow, pressure, and volume) of a rapidly inhaling human are captured during the simulation with high fidelity. Properties of the human respiratory system such as airway resistance and lung compliance are represented by using lung equivalents (porcine lungs) and mechanical UAMs (based on CT examinations). The displacement of gases during spontaneous breathing occurs due to the pressure difference between the atmosphere and the human lung. This physiological process is recreated by the xPULM™. During the breathing simulation, pressure changes in the thoracic chamber are induced by the movement of a bellows system. For inhalation, a negative pressure is created in the chamber by expanding the bellows, leading to air following the pressure gradient resulting in inflation of the lung equivalent. During exhalation, the opposite process occurs. The bellows is moved back to its original position, increasing the pressure in the chamber and deflating the lung equivalent. The movement of the bellows system can be precisely adjusted in the control software of the xPULM™. This allows for the simulation of different breathing scenarios under various conditions as demonstrated in [35]. A detailed description of the xPULM™ functionality and components including validation measurements are presented in our previous work [36].

#### 2.2.1. Representation of the Upper Respiratory Tract

**Figure 2:**
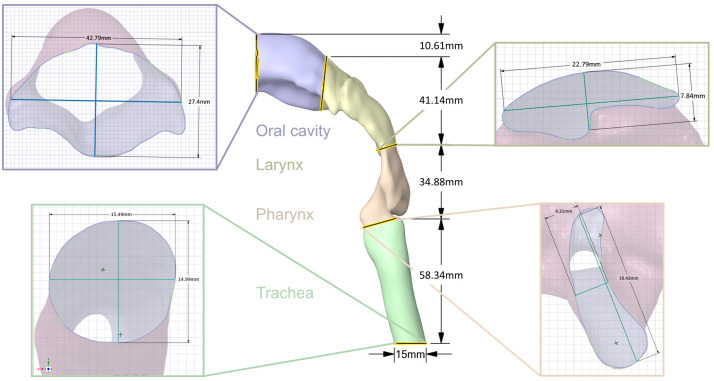
The manufactured 3D model of an upper respiratory tract of a 28-year-old, healthy, nonsmoking, male.

The mechanical UAM includes the oral cavity, oropharynx, larynx, and trachea. A CT examination of a 28-year-old, healthy, nonsmoking male was used for the UAM reconstruction. The subject has been annotated as healthy by clinical staff and did not show any sign of abnormal restrictions or geometrical limitations. Therefore, this CT examination has been considered to serve as a valid representation of an exemplary upper airway similar to previous works [37,38]. The selected dataset contained 280 images with a slice thickness of 0.75 mm and was exported in a Digital Imaging and Communications in Medicine (DICOM) format for further processing. The upper respiratory tract was segmented using a combination of thresholding and region growing techniques. The outcomes of the semiautomatic segmentation were inspected on a slide-by-slide basis and the segmentation parameters were adapted to obtain a precise segmentation of the upper airways. The resulting 3D model was exported as a Standard Triangle Language (STL) file and postprocessed to be 3D-printable. The final 3D mechanical UAM was manufactured using rapid prototyping techniques and coated with resin. An emphasis was placed on positioning the model to minimize usage of support structure in the flow path. The dimensions for each section of the final model are summarized in Figure 2 and Table 1. Custom connectors were designed based on the geometry of each DPI to ensure an airtight connection between the inhaler and the mechanical UAM.

All rapidly produced components were manufactured from polylactic acid (PLA) with a wall thickness of 2 mm and a layer height of 0.2 mm.

#### 2.2.2. Representation of the Lower Respiratory Tract

The lower respiratory tract consists of the bronchi, bronchioles, and alveoli, which form the lung. During breathing simulations, these structures have been represented by a primed porcine lung. The lung was salvaged from a slaughterhouse process and was therefore compliant with the 3R principles [39], which denote responsible use of animal or animal organs during experiments. The Nasco-guard^®^ (Nasco, WI, USA) preservation process kept the porcine lung inflatable, elastic, and covered with the parietal pleura. These properties are necessary for physiologically and anatomically realistic simulations of human breathing.

### 2.3. Dry Powder Inhalers

In total, four DPIs were evaluated in this study, grouped into single-dose and multiple-dose inhalers. The single-dose devices (BreezHaler^®^ and HandiHaler^®^) are loaded with a capsule containing the dose which is punctured prior to use. The remaining three were multidose DPIs (Ellipta^®^, Turbuhaler^®^), which store multiple doses within the devices. Summary of the relevant parameter values of the used DPIs is given in Table 2. Outlets of all DPIs were modified with custom rapidly manufactured adapters to enable a well-fitted, airtight connection to the oral cavity of the mechanical UAM.

### 2.4. Data Processing and Statistics

The optical aerosol spectrometry measurements were conducted with 128 intervals per decade. The arithmetic center of the intervals ($x_{i}$) is then:

(1) $$x_{i}=\;x_{i,lower}\;+\frac{x_{i,upper}\;-x_{i,lower}}{2}\;=\;x_{i,lower}\;+\frac{\Delta x_{i}}{2}\;\left[\mu m\right]$$

For further calculations, the differential particle number distribution $q_{0}\left(x_{i}\right)$ is defined as:

(2) $$q_{0}\left(x_{i}\right)=\;\frac{1}{\sum n_{i}}\;\frac{n_{i}}{\Delta x_{i}}\;\left[\mu m^{-1}\right]$$

where $n_{i}$ is the measured particle number within the interval limits $x_{i,lower}$ and $x_{i,upper}$. Leading to the mean particle diameter $M_{1}$ calculation:

(3) $$M_{1}=\sum \left(x_{i}\;q_{0}\left(x_{i}\right)\;\Delta x_{i}\right)=\;\bar{x}\;\left[\mu m\right]$$

Further information about the inhaled aerosol is obtained by calculating the particle number concentration:

(4) $$dCn=\;n_{i}\;\frac{1}{V_{m}}\;\left[{P/\mathrm{cm}}^{3}\right]$$

where the measured volume $V_{m}$ is defined as:

(5) $$V_{m}=u\;Iw\;t_{measurement}\;\left[{\mathrm{cm}}^{3}\right]$$

where *u* is particle velocity and Iw is the cross-section of the optical sensor.

For the chosen measurement setup, Equation (5) can be simplified to

(6) $$V_{m}=Qt_{measurement}\;\left[{\mathrm{cm}}^{3}\right]$$

where *Q* is the volumetric airflow (5 L/min). The measurement data is evaluated with nonparametric methods as the requirements for normal distribution and hence parametric test methods are not fulfilled. The data groups are compared pairwise using the Kruskal–Wallis test by ranks (or one-way ANOVA on ranks) with a significance level of α = 0.05; *H* values and *p* values are calculated and compared to a critical χ${}^{2}$ = 3.841 for a degree of freedom *df* = 1.

## 3. Results and Discussion

### 3.1. Inspiratory Flow Rate and Pressure Drop Measurements

**Figure 3:**
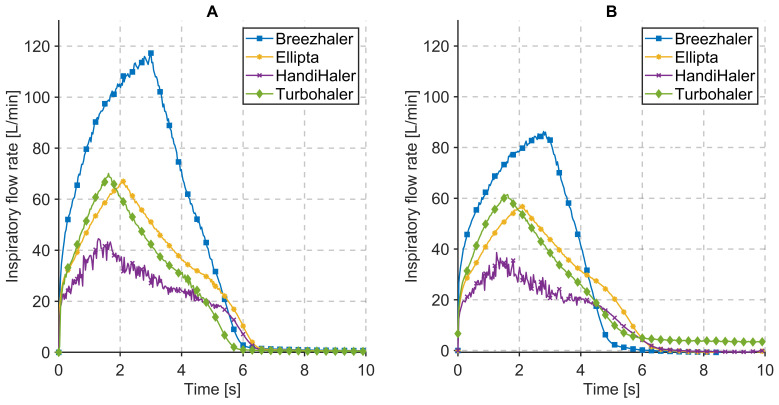
Flow profiles during (**A**) characterization measurements, (**B**) respiration measurements while inhaling through Breezhaler^®^, Ellipta^®^, HandiHaler^®^, and Turbohaler^®^ at a pressure drop given in Table 3.

Flow profiles measured during characterization and respiration measurements for the evaluated DPIs are presented in Figure 3. During characterization measurements, the resistance of the system is primarily resulting from the inner resistance of the DPIs. The peak inspiratory flow, measured at the pressure drop values, provided in Table 3, ranges from 45 to 120 L/min. The shape of the inhalation profile, shown in Figure 3A, is characteristic for each used DPIs and reflects the individual constructional solution of the devices included in this evaluation. Vibrations of the capsule, for example, are distinctive for HandiHaler^®^ and manifest in rapid oscillations of the inspiratory flow. Inhalation time required to reach the necessary pressure drop is influenced by the inner resistance of the DPIs. The shape of the measured flow profiles with xPULM™are comparable to full flow rate profiles of patients [40].

The inspiratory flow rate during respiration measurements is, in contrast to characterization measurements, influenced by resistance and compliance of the included mechanical UAM and the primed porcine lung, respectively. This is evident for DPIs with low inner resistance (e.g., Breezhaler^®^) where the inspiratory flow rate drops by 30 L/min. In case of DPIs with higher inner resistance (e.g., HandiHaler^®^), the flow rate is influenced moderately as the increase of the overall system resistance is lower. The peak inspiratory flow, measured at the pressure drop values, provided in Table 3, ranges from 39 to 86 L/min. The system resistance refers to a combination of DPI inner resistance (constant) and the resistance of attached pneumatic components.

**Figure 4:**
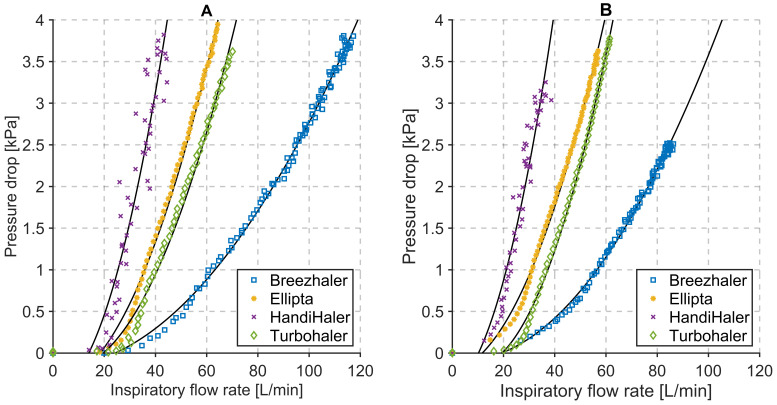
Relationships between inspiratory flow rate and pressure drop of four commercial DPIs during (**A**) characterization measurements and (**B**) respiration measurements.

The flow results of the different DPIs, as shown in Figure 3, allow conclusions on the handling of the inhaler and its characteristics during use. The HandiHaler^®^, for example, shows a wider range of flow values as well as higher volatility in pressure drop values (see Figure 4), than most of the other inhalers. This is mainly caused by the propelling mechanism, which is based on the mechanical movement of the aerosol-loaded capsule. Based on the user guide, the capsule has to move (also acoustically noticeable) within the inhaler in order to disperse the powder. This oscillating movement leads to a volatile flow and oscillating pressure drop measurements; therefore, characterization of this inhaler is influenced by the handling of the capsule and inhaler.

A comparable observation can be made for the use of the second capsule-based DPI, the Breezhaler^®^. This product is also based on the oscillation of the capsule in order to propel the aerosol properly. These oscillations are moreover influenced by the holding position and angle of the device during inhalation. In contrast to the HandiHaler^®^, the capsule within the Breezhaler^®^ is not limited in movement mechanically but mainly by gravitation. When the Breezhaler^®^ is moved to a horizontal position the likelihood of the capsule dropping out of the holding cavity increases, impacting the aerosol production mechanism.

The correspondingly changed flow profiles caused by different lung equivalents can be observed in Figure 3. The compliance of the introduced lung tissue (depicted by the flow curves in Figure 3B) influences the peak flow as well as the flow profile. The anatomic components of the used porcine lung and its geometric properties lead to a prolonged inhalation time and flattened flow profile when using identical inhalation settings as with the polymer-based breathing bags as lung equivalent.

### 3.2. Influence of the Mechanical UAM and the Primed Porcine Lung

Effects of the mechanical UAM and the primed porcine lung during respiration measurements are evident from the relationship between inspiratory flow rate and pressure drop across the inhalers (see Figure 4B). The resistance of the measurement system increases significantly (*p* < 0.05) with all inhalers (see Table 3) and a pressure drop of 4 kPa is reached for lower inspiratory flow rates.

The measurements revealed limitations in reaching the pressure drop of 4 kPa consistently for Breezhaler^®^. Based on the recorded observations, the position of the capsule within the DPI and the angle of the device are critical. Even a slight movement of the capsule changes the behavior of the device. The pressure drop set prior to measurements could not be reached despite high inspiratory flow and prolonged inhalation time. A pressure drop ≥1 kPa with any DPI is sufficient for the patient to receive an adequate lung dose [9]. This criterion (defined as a minimum requirement) was met over all conducted measurements.

Relevant parameter values for the used DPIs, characterization measurements, and respiration measurements are summarized in Table 3. These parameter values complement the graphical result shown in Figure 3 and Figure 4. Additionally, they provide further inside about the relationship between the inner resistance of the DPIs, inhaled volume, inhalation time, and peak inspiratory flow at particular pressure drop values.

The difference between the inner resistances of DPI measured during characterization and the values extracted from the literature is in an acceptable tolerance range of ± 0.01 kPa${}^{1/2}$/L/min.

### 3.3. Changes in Mean Particle Diameter

**Figure 5:**
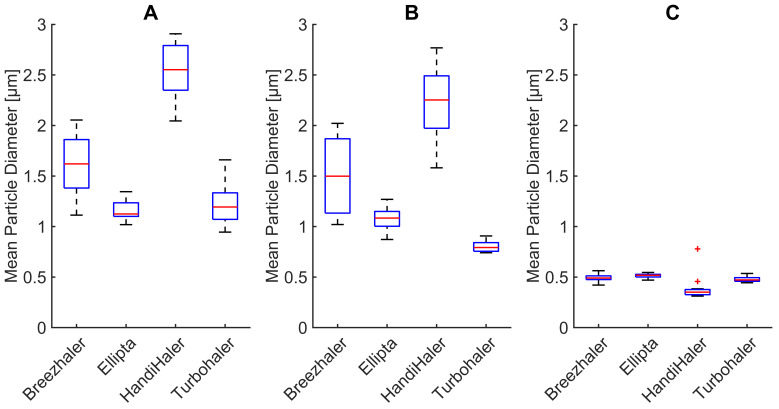
Changes in mean particle diameter during (**A**) characterization measurements, (**B**) inhalation measurements, and (**C**) exhalation measurements for four commercial DPIs.

Changes in mean particle diameter ($M_{1}$) during DPI characterization and respiration measurements using the mechanical UAM and primed porcine lung are depicted in Figure 5. During characterization measurements, the mean particle diameter ranges from 0.95 μm (TurboHaler^®^) to 2.90 μm (HandiHaler^®^). These results are comparable to literature values reporting particles ranging from 2.20 μm (Ellipta^®^) to 3.90 μm (HandiHaler^®^) [40]. Differences in the absolute values of mean particle diameter are to be expected, based on the different components of the used measurement setup. As reported by several authors [9,10,40,41], the aerodynamic properties of the generated drug particles vary based on quantities such as peak inspiratory flow rate, flow acceleration, inhalation time, and inhaled volume. These quantities are patient-specific and vary from the presented measurements. Filtration properties of the mechanical UAM cause the mean particle diameter to shift toward lower values during inhalation. This can be observed for all tested DPI, as Figure 5 depicts. It has been shown that the upper respiratory tract indeed acts as a particulate filter. Larger particles (>3 μm) deposit more easily in the upper respiratory tract, whereas the smaller particles (<3 μm) pass into the lower respiratory tract as the filtration function decreases with particle size [42,43].

Exhaled particles during our measurements are characterized by a mean particle diameter in a narrow range from 0.31 μm (HandiHaler^®^) to 0.56 μm (BreezHaler^®^). These results were expected as the deposition of aerosol particles in the lung reaches its minimum at 0.5 μm [44,45]. Furthermore, there is a significant difference (*p* < 0.05) in mean particle diameter between inhaled and exhaled particles Figure 5B,C for all tested DPIs (K-W test, H = 17.29, *p* = 0.00003). This change is caused by the interaction of the aerosol particles with the primed porcine lung tissue. The interaction is caused by a highly complex and constantly changing inner geometry of the lung tissue, which influences the mean particle diameter. Additionally, the high relative humidity within the lung tissue may lead to hygroscopic growth and therefore also to adhesion of particles.

### 3.4. Deposition of Particles in the Porcine Lung

**Figure 6:**
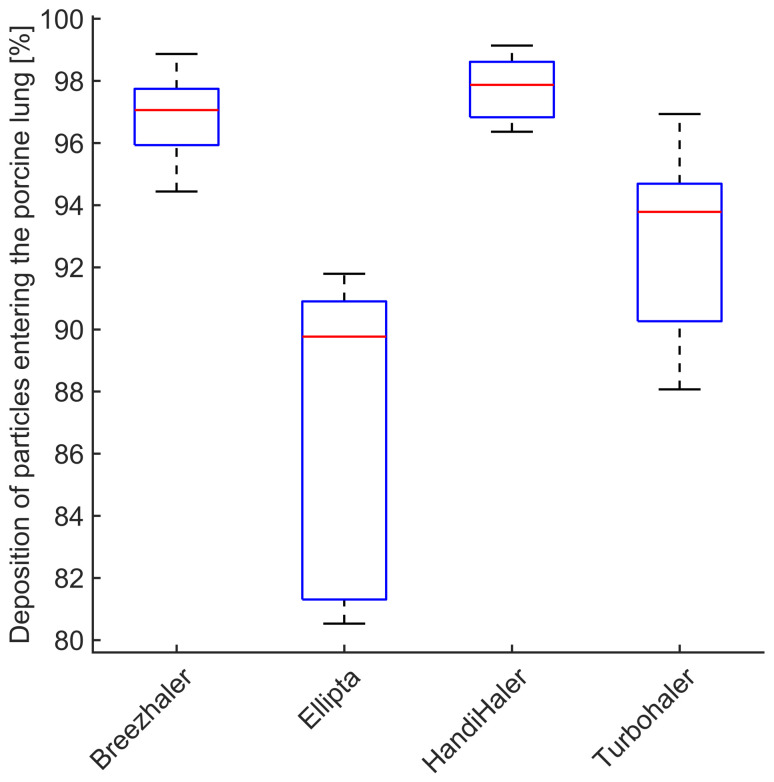
Deposition of aerosol particles in the porcine lung (expressed as a percentage of particle number concentration measured posterior of the mechanical UAM) for four commercial DPI inhalers.

**Figure 7:**
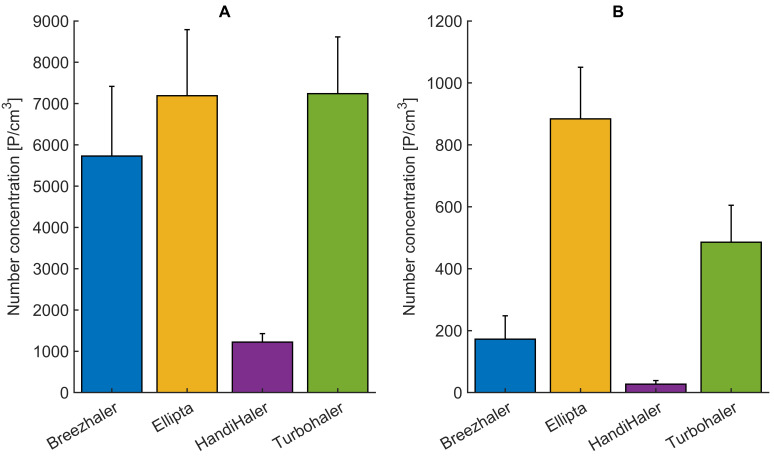
Differences between aerosol particle number concentration sampled from the air stream during (**A**) inhalation and (**B**) exhalation for four commercial DPIs. Respiration parameters are provided in Table 3.

The difference between the particle number concentration in inhaled and exhaled air can be considered as number concentration of particles depositing in the porcine lung. The deposition is expressed as a percentage of particle number concentration averaged over the individual inhalation or exhalation cycles and depicted in Figure 6. The deposition of particles in the respiratory tract reaches its minimum in the range of 0.40 μm–0.60 μm [45]. The measured particle size distribution for Ellipta and Turbohaler during inhalation is characterized by lower mean particle diameters ( 1.08 μm and 0.79  μm, respectively). This corresponds to the deposition effects and measured number concentration represented in Figure 6 and Figure 7. However, all measured DPIs show deposition above 80% achieving the intended drug delivery.

Differences between aerosol particle number concentration sampled from the air stream during (A) inhalation and (B) exhalation for all inhalers are depicted in Figure 7. There is a statistically significant difference (*p* < 0.05) between particle number concentration in inhaled and exhaled airstream for all tested inhalers (K-W test, H = 17.29, *p* = 0.00003). This is caused by particles depositing in the primed porcine lung.

The generated drug particles from the DPIs are inhaled through the mechanical UAM, which represents the nasooropharyngolaryngeal region (extrathoracic region). Larger particles (>3 μm) deposit in this region mainly due to effects of inertial impaction [45]. The rest of the drug particles penetrates the deeper regions of the respiratory tract model and reach the primed porcine lung. The complex geometry and high relative humidity of the lung present an ideal environment for most of the particles to deposit due to sedimentation and Brownian diffusion [22,45].

Regional lung deposition and bronchodilator response of pharmaceutical aerosols was studied extensively in previous works [46,47]. Their results confirm that small particles are exhaled with exhalation fractions for particle diameters 1.5 μm, 3 μm and 6 μm being 22%, 8%, and 2%, respectively [47]. A lung deposition study in healthy human subjects showed a exhalation fraction of exhaled dose of 1.2% [48]. In this study, however, a MAGhaler DPI was used to aerosolize the powder.

Research conducted with healthy individuals, asthmatic, and COPD patients show no significant difference in drug deposition of aerosols generated with DPIs [49]. The reported fraction of exhaled particles ranges between 1.6% and 3.3%. These findings are consistent with our measurements where the fraction of exhaled particles ranges from 2.13% (HandiHaler^®^), 2.94% (BreezHaler^®^), and 6.22% (Turbohaler^®^) to 10.24% (Ellipta^®^).

## 4. Summary and Conclusions

For a large number of patients, DPIs are the device of choice for the delivery of pharmaceuticals to manage asthma and COPD [10,50]. The number of commercially available DPIs is growing [9] with inhalers varying in their design, operating mechanisms, and resistance to inhaled airflow [9,51]. Accounting for these properties and the patient’s ability to use the specific device is essential for efficient drug delivery. Testing setups provide an option to evaluate aerosolized dry powders generated by DPIs and allow for further insights into DPI performance under various conditions [19,52,53,54].

In this work, aerosol particle diameter and particle number concentration of pharmaceutical aerosols generated by four commercially available DPIs were investigated. The measurement setup consists of the active respiratory system model xPULM™ in combination with optical aerosol spectrometry and a mechanical UAM. This allows for the evaluation of pharmaceutical aerosols in the range of 0.2 μm to 10 μm and the calculation of deposition of particles in the porcine lung under realistic inhalation and exhalation simulations. Experimental data measured during exhalation are scarce when in vitro pharmaceutical aerosol test systems are employed due to the operating principle of impactors.

To represent the human upper respiratory tract with high fidelity a mechanical UAM was developed, manufactured, and introduced as a part of the xPULM™. The model was derived from CT examinations of a 28-year-old healthy male, which has been clinically annotated. A primed porcine lung was used to simulate the complex inner structures of the human lower respiratory tract. The integration of the mechanical UAM and primed porcine into the xPULM™ model represents an important step forward towards the realistic simulation of a breathing human. Additionally, the combination of xPULM™ with an optical aerosol spectrometer presents an alternative approach to animal experimentation suitable for applications in aerosol research.

Our results can be summarized as follows:

- Integration of a mechanical UAM, as a part of the xPULM™, increases the resistance of the overall system. This affects inhalatory flow and pressure characteristics of DPIs with lower inner resistance more than DPIs with high inner resistance, where the change is negligible.
- Inclusion of a porcine lung as a representation of the human lower respiratory tract (compliant with the 3R principles) allows comparable particle deposition to reported findings [47,48,49].
- Handling and placement of a capsule into single-dose DPIs influences aerosol production during inhalation drug therapy. Slight changes in capsule placement may influence the amount of delivered drug. Correct handling of the inhaler should be emphasized alongside acceptable inhalation maneuvers (as defined by the device manufacturer) to ensure the desired result.
- Mean particle diameter is reduced by the filtration properties of the mechanical UAM, affecting mostly larger particles (>3 μm). Such models, when based on CT examinations, are reliably representing the function of the human upper respiratory tract.
- The majority of particles entering the porcine lung deposit within minimum deposition is reached for the particle size of (0.5 μm). The primed porcine lung is therefore a suitable lung equivalent and representation of the human lung.
- Sampling of the airstream during inhalation and exhalation and its subsequent evaluation using optical aerosol spectrometry techniques is a viable alternative to impactors for evaluating pharmaceutical aerosols.

In conclusion, the xPULM™ active respiratory system model in combination with the introduced mechanical UAM and the optical aerosol spectrometer is a viable option for investigating particle diameter and particle number concentration of pharmaceutical aerosol depositing in the porcine lung under realistic breathing conditions. Further research will focus on the inclusion of additional components and techniques (e.g., nanodots, tissue sampling, histopathology) to quantify the regional deposition of pharmaceutical aerosols in lung tissue obtained by 3R compliant processes. Additionally, coating of the inner surface of the mechanical UAM will be considered, to ensure the least possible artifacts and interference of the 3D printing materials on the particle transportation effects. Besides regional deposition, mass-based approaches will also be included to further increase comparability with established deposition measurement techniques.

## Figures and Tables

**Table 1:** Summary of the mechanical UAM dimensions of a 28-year-old, healthy, nonsmoking, male. The sections correspond to the highlighted sections in Figure 2. SA—surface area.

Section	Volume [mm${}^{3}$]	Lower SA [mm${}^{2}$]	Diameter Y [mm]	Dimeter X [mm]	Upper SA [mm${}^{2}$]	Diameter Y [mm]	Diameter X [mm]
Trachea (green)	10,657.02	188.39	15.49	14.99	86.60	18	4.93
Pharynx (orange)	7311.52	119.06	19.43	4.31	86.60	4.86	24.51
Larynx (yellow)	15,902.39	119.11	7.84	22.79	777.57	24.93	44.34
Oral cavity (blue)	22,265.60	777.60	7.84	22.79	529.90	7.84	22.79

**Table 2:** Summary of the relevant parameter values of the used DPIs taken from the literature [9,40].

Device	Active Substance	Resistance [kPa^1/2^/L/min]	Metered Dose [μg]	Lactose [mg]	Dose Type
Seebri^®^ Breezhaler^®^	Glycopyrronium	0.0216	44	23.6	multidose, predispensed
Anoro^®^ Ellipta^®^	Fluticasone furoate and vilanterol	0.0286	55/22	25	multidose, predispensed
Spiriva^®^ HandiHaler^®^	Tiotropium bromide	0.0504	18	5.5	single-dose, hard capsules
Symbicort^®^ Turbohaler^®^	Budesonide and formoterol	0.0313	200/6	0.73	multidose, predispensed

**Table 3:** Summary of the relevant parameter values for the used DPIs during characterization and respiration measurements. V_INH_—inhaled volume, P_DROP_—pressured drop across the inhaler during inhalation, PIF—peak inspiratory flow, and t_INH_—inhalation time.

Dry Powder Inhalers	Characterization Parameters					Respiration Parameters				
Device	Vinh [L]	P_DROP_ [kPa]	PIF [L/min]	Tinh [s]	System Resistance [kPa^1/2^/L/min]	Vinh [L]	P_DROP_ [kPa]	PIF [L/min]	Tinh [s]	System Resistance [kPa^1/2^/L/min]
Seebri^®^ Breezhaler^®^	6.98	3.81	117.28	3.00	0.0166	4.55	2.52	86.31	3.00	0.0184 *
Anoro^®^ Ellipta^®^	4.06	4.27	67.01	2.10	0.0308	3.38	3.63	56.94	2.10	0.0335 *
Spiriva^®^ HandiHaler^®^	2.60	3.82	44.50	1.40	0.0439	2.05	3.25	38.62	1.40	0.0467 *
Symbicort^®^ Turbohaler^®^	3.52	3.83	70.11	1.60	0.0279	3.40	3.89	61.48	1.60	0.0320 *

* *p* < 0.05 for difference between resistances measured with and without the mechanical UAM.

## Data Availability

The data that support the findings of this study are available upon reasonable request.
